# Correction to “G protein pathway suppressor 2 suppresses gastric cancer by destabilizing epidermal growth factor receptor”

**DOI:** 10.1111/cas.16391

**Published:** 2024-11-05

Si Y, Zhang H, Peng P, et al. G protein pathway suppressor 2 suppresses gastric cancer by destabilizing epidermal growth factor receptor. *Cancer Sci*. 2021;112(12):4867–4882.

The authors regret that the images in Figure 3J were inadvertently misused during figure preparation. The correct images are shown below.

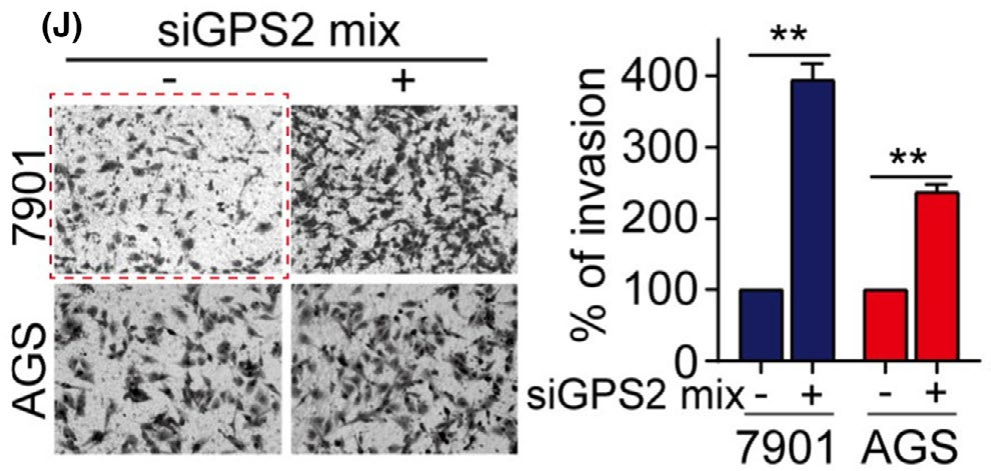

While saving the images from the transwell invasion experiment with SGC7901 cells overexpressed GPS2 (Figure 3F), the images were inadvertently named and placed in the folder for the transwell invasion experiment with SGC7901 cells transfected with NC siRNA (Figure 3J). As a result, the overexpressed GPS2 group image from the transwell invasion experiment with SGC7901 cells in Figure 3F was inadvertently reused in Figure 3J.

We apologize for this error.

